# Does sex-biased dispersal account for the lack of geographic and host-associated differentiation in introduced populations of an aphid parasitoid?

**DOI:** 10.1002/ece3.1504

**Published:** 2015-05-06

**Authors:** Francisca Zepeda-Paulo, Blas Lavandero, Frédérique Mahéo, Emilie Dion, Yannick Outreman, Jean-Christophe Simon, Christian C Figueroa

**Affiliations:** 1 Laboratorio de Interacciones Insecto-Planta, Instituto de Ciencias Biológicas, Universidad de Talca2 Norte 685, Talca, Chile; 2 Facultad de Ciencias, Universidad Austral de ChileIndependencia 641, Valdivia, Chile; 3 Millennium Nucleus Centre in Molecular Ecology and Evolutionary Applications in the Agroecosystems2 Norte 685, Talca, Chile; 4 INRA, Institut de Génétique, Environnement et Protection des Plantes (UMR IGEPP), Domaine de La Motte35653, Le Rheu Cedex, France

**Keywords:** Aphid parasitoids, *Aphidius ervi*, genetic differentiation, sex-biased dispersal, host fidelity, microsatellite loci, sibship inference

## Abstract

Host recognition and use in female parasitoids strongly relies on host fidelity, a plastic behavior which can significantly restrict the host preferences of parasitoids, thus reducing the gene flow between parasitoid populations attacking different insect hosts. However, the effect of migrant males on the genetic differentiation of populations has been frequently ignored in parasitoids, despite its known impact on gene flow between populations. Hence, we studied the extent of gene flow mediated by female and male parasitoids by assessing sibship relationships among parasitoids within and between populations, and its impact on geographic and host-associated differentiation in the aphid parasitoid *Aphidius ervi*. We report evidences of a high gene flow among parasitoid populations on different aphid hosts and geographic locations. The high gene flow among parasitoid populations was found to be largely male mediated, suggested by significant differences in the distribution of full-sib and paternal half-sib dyads of parasitoid populations.

## Introduction

In Hymenopteran parasitoids, host choice strongly relies on host fidelity, a plastic behavior which can significantly restrict the host preferences of parasitoids, resulting in a lower dispersion and gene flow between populations associated to certain hosts (Feder et al. 1993, 1994; Henry et al. 2008). Host fidelity refers to individual female parasitoids showing a learned preference to deposit eggs in hosts of the same species from which they themselves emerged (Turlings et al. 1990, 1993; Vet and Dicke 1992), a behavior that could be developed through associative learning (Lewis and Tumlinson 1988), and which has been hypothesized to occur by genetic imprinting through epigenetics inheritance of phenotypically plastic traits (Davis and Stamps 2004).

In this sense, several studies have shown the existence of host fidelity to different aphid–plant complexes by females of the aphid parasitoid *Aphidius ervi* (Daza-Bustamante et al. 2002; Henry et al. 2008; Zepeda-Paulo et al. 2013); conversely, little is known on the male preference for mating. Host fidelity has been implicated in the rapid experimental evolution of virulence (a proxy of parasitoid fitness) of *A. ervi* on novel hosts (Henry et al. 2008) and during the rapid genetic divergence in experimental populations of *A. ervi* reproduced on different host species (Emelianov et al. 2011). Such host-induced behavioral mechanism could generate nonrandom dispersal, thus reducing the gene flow between parasitoid populations attacking different insect hosts (Emelianov et al. 2011; Feder and Forbes 2010). However, the effect of migrant males on genetic differentiation of populations has been frequently ignored in parasitoids, despite its putative impact on gene flow between parasitoid populations (Henry 2008). In fact, evidences of a reduction in patch residence time of *A. ervi* males on natal host patches compared with that of females has been reported, which encourages male dispersion from the natal host patch (Nyabuga et al. 2012). On the other hand, the copulatory experience along with the olfactory stimuli derived from host–plant complexes present during the parasitoid copulation could affect by subsequent mate searching of *A. ervi* males (Villagra et al. 2008). Furthermore, evidence of assortative mating strongly influenced by the body size of males and linked to the natal aphid host has been also reported in *A. ervi* (Henry 2008). However, the extent to which male parasitoid gene flow affects geographic and host-associated differentiation in parasitoid populations has never been addressed.

Consequently, our goal was to estimate the extension which male and female gene flow influences genetic differentiation between different populations in the parasitoid *A. ervi*. We characterized the genetic diversity and population structure of the introduced populations of *A. ervi* in Chile, estimating the gene flow between populations collected on different hosts and geographic locations in the field and assessing the occurrence of genetic differentiation associated with (1) three different aphid species (interspecific level), including the cereal aphids *Sitobion avenae* Fabricius and *Rhopalosiphum padi* Linnaeus, and the pea aphid *A. pisum*; and (2) three different host races of *A. pisum* (intraspecific level). Only weak signals of genetic differentiation were found, both at the interspecific and intraspecific levels. Therefore, to estimate and compare the extent of female and male-mediated gene flow between parasitoid populations, a sibship reconstruction was performed.

## Materials and Methods

### Sampling parasitoids

**Figure 1:**
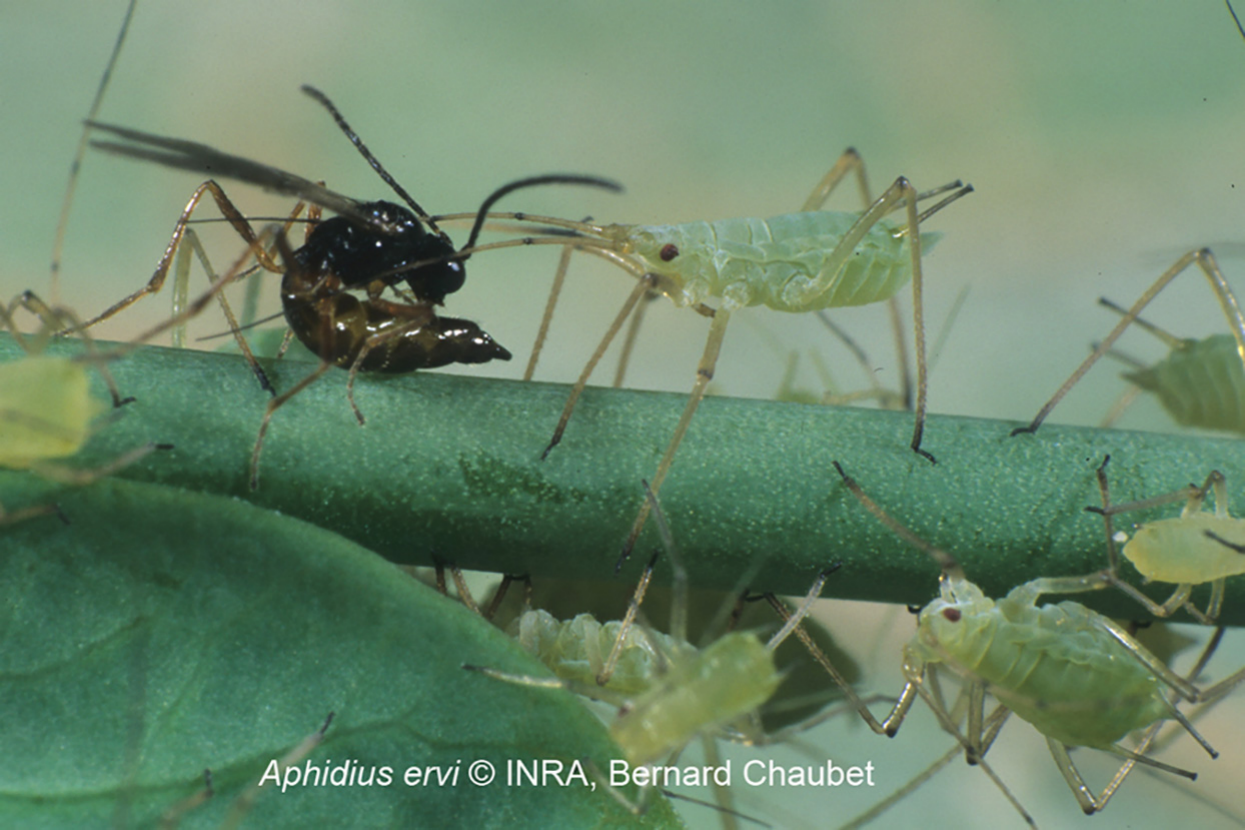
The parasitoid *Aphidius ervi* attacking a pea aphid.

Populations of *A. ervi* in Chile were introduced from France during the mid-1970s, as part of a cereal aphid biocontrol program mainly to control the grain aphid *S. avenae*, and is presently one of the best examples of biocontrol of *S. avenae* and *A. pisum* (Gerding and Figueroa 1989; Gerding et al. 1989; Starý 1993; Starý et al. 1993; Zepeda-Paulo et al. 2013). Populations of *A. ervi* in Chile show a similarly high virulence (with no fitness trade-offs) and host fidelity in parasitoid females on both *S. avenae* and *A. pisum* complex (alfalfa and pea host races) (Zepeda-Paulo et al. 2013) (Fig.1).

**Figure 2:**
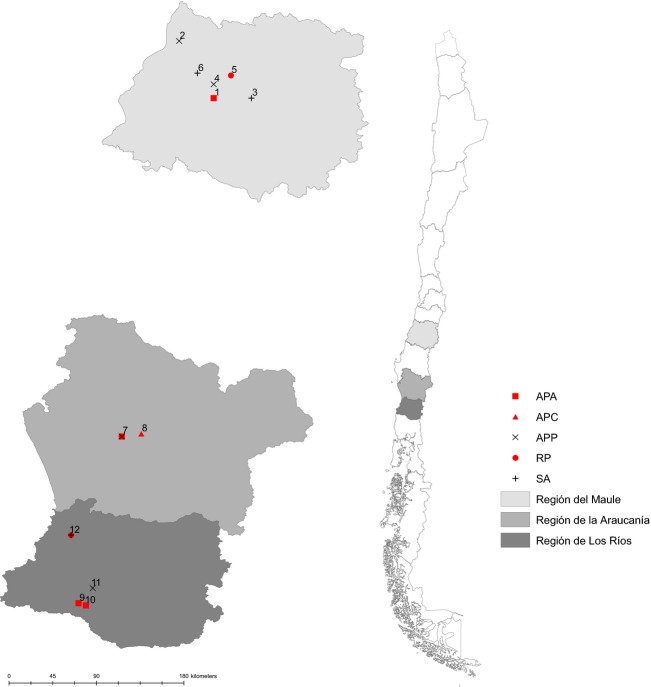
Localities and aphid hosts where populations of *Aphidius ervi* were sampled. Each parasitoid population was sampled on different aphid hosts (APA:*A. pisum* alfalfa; APP:*A. pisum* pea; APC:*A. pisum* clover; SA:*S. avenae* and RP:*R. padi*) in different locations (different numbers) in two geographic zones in Chile: Central (Maule Region) and South (La Araucanía and Los Ríos regions). 1: Colin (CO), 2: Curepto (CU); 3: San Clemente (SC), 4: Tinajas (TT), 5: Panguilemo (PT), 6: Tejerías (TE), 7: Vilcún-1, 8: Vilcún-2, 9: La Unión (U), 10: Puerto Nuevo (PN), 11: Paillaco (VP), and 12: Pichoy (PI).

Parasitoid individuals were obtained by collecting live aphids from cereal and legume crops in central and southern Chile during two consecutive crop seasons (2010/2011 and 2011/2012) (Fig.2). The grain aphid, *S. avenae*, and the bird cherry-oat aphid, *R. padi*, were both collected on wheat and oat fields. The three commonest host races of the pea aphid, *A. pisum* (specialized on alfalfa, pea–lentil and clover, respectively) (Peccoud et al. 2008, 2009; Peccoud and Simon 2010), were collected on alfalfa, pea, lentil, and red clover fields (Table1). The study area included several farms from two agroclimatic regions including central Chile (Maule Region) and southern Chile (La Araucanía and Los Rios regions), geographic zones which are characterized by contrasting climates: dry Mediterranean and temperate rainy, respectively (Fig.2 and Table1). Aphid species were determined following taxonomic keys (Blackman and Eastop 1984). All aphid individuals collected in the field were then transferred to the laboratory for rearing and parasitoid emergence. This sampling method allowed us to unequivocally determine the species of aphid host from which each parasitoid individual emerged. To ensure a representative sample of the genetic diversity in each field, parasitoid populations were sampled at each locality on intervals of 10 m between each plant (Antolin et al. 2006). Aphids were reared on the same plants on which they were sampled in the field, under controlled conditions in the laboratory (20 ± 1°C; 50–60 RH; L16:D8). These conditions assured the successful development of parasitoids. The formation of aphid mummies (the distinctive shape of exoskeleton when a dead aphid contains a parasitoid pupa) and the subsequent emergence of adult parasitoids were observed during the following days. All parasitoid individuals emerging from the mummies were sexed, determined to the species level utilizing a key to Aphidiinae parasitoids described by Starý (1995), and preserved in 95% ethanol until DNA extraction. Finally, since *A. ervi* is a haplodiploid species (i.e., diploid females come from fertilized eggs, while haploid males origin from unfertilized eggs), only diploid females were used for genotyping to ease population genetic analyses and sibship reconstructions (Nyabuga et al. 2011).

**Table 1:** Sampling information. The locations and aphid hosts where *Aphidius ervi* parasitoid individuals were sampled are indicated for the Maule and La Araucanía/Los Rios regions, specifying the season when they were sampled

Region	Aphid host	Plant	Location	Code	Season	Lat. S	Long. W
Central	APA	Alfalfa	Colin	CO	2010/2011	−35.47	−71.75
	APP	Lentil	Curepto	CU	2010/2011	−35.05	−72.07
	SA	Wheat	San Clemente	SC	2010/2011	−35.58	−71.4
	APA	Alfalfa	Colin	CO	2011/2012	−35.47	−71.75
	APP	Pea	Tinajas	TT	2011/2012	−35.45	−71.75
	RP	Oat	Panguilemo	PT	2011/2012	−35.37	−71.59
	SA	Wheat	Tejerías	TE	2011/2012	−35.35	−71.90
South	APA	Alfalfa	Vilcún 1	V1	2010/2011	−38.71	−72.51
	APC	Clover	Vilcún 2	V2	2010/2011	−38.69	−72.42
	APP	Pea	Vilcún 1	V2	2010/2011	−38.71	−72.51
	APA	Alfalfa	La Unión	U	2010/2011	−40.26	−73.0
	APA	Alfalfa	Puerto Nuevo	PN	2011/2012	−40.28	−72.89
	APP	Pea	Paillaco	VP	2011/2012	−40.12	−72.87
	SA	Oat	Pichoy	PI	2011/2012	−39.63	−73.07
	RP	Oat	Pichoy	PI	2011/2012	−39.63	−73.07

The geographic coordinates are also listed. APA: *Acyrthosiphon pisum* alfalfa race; APP: *A. pisum* pea–lentil race; APC: *A. pisum* red clover race; SA: *Sitobion avenae*; RP: *Rhopalosiphum padi*.

### Development of microsatellite markers for *Aphidius ervi*

New microsatellite markers for *A. ervi* were developed from an equimolar mixture of DNA isolated from 20 individuals. The DNA was screened for microsatellite loci using 454-pyrosequencing (454 GS-FLX Titanium, Roche Applied Science), isolating single sequence repeats (SSR) from an enriched library. The procedure was carried out through the Ecomicro Consortium (Malausa et al. 2011). Among the hundreds of loci found, putative microsatellite markers were selected on the basis of short and simple motifs, and motif repeat number (≥5), which were set up as the main criteria to look for microsatellite sequences and their flanking sequences using the Primer 3 software (Untergasser et al. 2012). To get reliable and easy to score microsatellites, 48 SSR loci were assessed and sorted according to the quality of amplification and to the quality of their profiles after visualization of amplicons on a capillary sequencer. Among these, the best subset of 12 loci was selected for further studies. These 12 selected loci were first validated in simplex reactions, and three annealing temperatures were tested for each pair of primers to select the one providing the most reliable and reproducible amplification. The microsatellite markers thus sequenced and selected were named (Ae01, Ae03, Ae06, Ae08, Ae16, Ae20, Ae22, Ae27, Ae29, Ae30, Ae32, and Ae38). Primer sequences and diversity indexes for each locus are shown in Table S1. Sequences of microsatellite loci were deposited in GenBank (see at availability of supporting data). Null alleles, scoring errors due to stuttering, and large allele dropouts at the 12 microsatellite loci were tested using Microchecker v.2.2.3 (van Oosterhout et al. 2004). All 12 loci were polymorphic, ranging from 4 to 15 alleles per locus (Table S1). Total expected heterozygosity of each locus ranged from 0.412 to 0.763 (Table S1). Most loci were in Hardy–Weinberg equilibrium (HWE) based on inbreeding coefficients. However, four loci (Ae03, Ae20, Ae22, and Ae38) evidenced an excess of homozygotes (Table S1), which could be a consequence of null alleles for loci Ae20, Ae22, and Ae38, as suggested by the analysis on Microchecker (data not shown). Similarly, four of the fifteen sampled populations showed a significant presence of null alleles for Ae20, while nine populations evidenced null alleles for loci Ae22 and Ae38. Consequently, these three loci were excluded from further analyses.

### Parasitoid genotyping

DNA extraction was performed using a salting out method (Sunnucks and Hales 1996). Microsatellite loci were amplified by PCR using fluorescence labeled primers. Each PCR amplification was prepared in a 25 *μ*L reaction volume containing 1 *μ*L genomic DNA, 1× PCR buffer (20 mmol/L Tris-HCl, 50 mmol/L KCl), 2 mmol/L MgCl2, 0.2 mmol/L of each dNTP, 0.25 mmol/L of reverse primer, 0.25 mmol/L of M13 primer, 0.25 mmol/L of forward primer, and 0.5 U of Taq polymerase (Hufbauer et al. 2001). Electrophoresis was performed in a capillary sequencer (ABI 3130xl; Applied Biosystems, Foster City, CA, USA). Finally, the multilocus genotype for each parasitoid individual was obtained by uniting the allele combinations from all microsatellite loci after reading the sequencing electropherograms using the GENEMARKER software (Softgenetics, State College, PA, USA).

### Data analysis

#### Genetic variation within populations

The polymorphism at each locus and the genetic diversity for each population were calculated using Genalex v.6.5 (Peakall and Smouse 2012), examining the total and effective number of alleles, the allelic richness, the observed and expected heterozygosities, and the inbreeding coefficient (*F*_IS_). The term “parasitoid population” is used here operationally to describe a group of parasitoid individuals based on their ecological (different hosts), spatial (different regions), and temporal (different seasons) distributions in the field. Allelic richness was computed and standardized by the multiple random reduction method (Leberg 2002) using the smallest sample size (*n* = 12), with the package standArich v.1.0 in R (R Development Core Team 2011). Deviations from the HWE and linkage disequilibrium (LD) between pairs of loci were tested for each microsatellite locus and each population using GENEPOP v 3.4 (Raymond and Rousset 1995).

#### Genetic differentiation among parasitoid populations

The statistical power to detect low levels of genetic differentiation with the observed size samples, number of loci and number and frequency of alleles in the parasitoid populations under study was estimated using POWSIM 4.1 (Ryman and Palm 2006). This program simulates the estimated degree of genetic differentiation that could be detected on a particular set of loci using Fisher's exact test on all loci simultaneously. A total of 1000 simulation runs were carried out using sample sizes and allele frequencies of parasitoid populations under study, and it was assumed an effective population size (*N*_e_) of 1000 individuals and 10 of drift generations (*t*) to achieve expected *F*_ST_ values <0.005. The *F*_ST_ (fixation index) was estimated to determine the degree of genetic differentiation at the population level; an unbiased estimator of *F*_ST_, the Hedrick's standardized *G*_ST_ (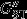), was also computed to further correct for bias when population sizes were small (Meirmans and Hedrick 2011). Both *F*_ST_ and 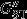 were calculated between pairs of parasitoid populations coming from different host races/species, regions, and seasons using Genalex v.6.5 (Peakall and Smouse 2012). Partitioning of genetic diversity was studied at three hierarchical levels performing three analyses to study the variation at the level of aphid hosts, regions, and seasons, with Genalex v.6.5 (Peakall and Smouse 2012). To study the most probable number of clusters (*k*) containing the different multilocus genotypes of *A. ervi* identified, as well as to compute the coefficient of ancestry for each multilocus genotype to the inferred different clusters, we performed a Bayesian assignment analysis using STRUCTURE v. 2.3.4 (Pritchard et al. 2000) on the entire dataset (676 multilocus genotypes from different aphid hosts, regions, and seasons) (Table2). We used the admixture and correlated allele frequencies models without any a priori geographic information. The number of clusters (*k*) with the highest posterior probability after 10 replicates for each *k* (between 1 and 10) was determined. The model was tested by performing 100,000 runs with 10,000 burn-in periods before each run. Finally, we assessed the most probable number of clusters using the log of the posterior probability and the average rate of change (delta *k*) for each value of *k*, using the method proposed by Evanno et al. (2005) implemented in Structure Harvester v.6.93 (Earl and von Holdt 2012).

**Table 2:** Genetic diversity in each parasitoid population according to the aphid host, sampled region and season

Region	Aphid host	Location	Season	*N*	*N*_e_ (SE)	A (SD)	*H*_o_ (SE)	*H*_e_ (SE)	u*H*_e_ (SE)	*F*_IS_ (SE)	LD
Central	APA	CO	2010/2011	48	3.061 (0.30)	4.82 (0.2)	0.652 (0.04)	0.646 (0.04)	0.652 (0.04)	−0.010 (0.02)	3/36
	APP	CU	2010/2011	92	3.168 (0.26)	4.97 (0.24)	0.657 (0.04)	0.663 (0.03)	0.666 (0.03)	0.009 (0.02)	0/36
	SA	SC	2010/2011	41	3.009 (0.29)	4.78 (0.25)	0.637 (0.04)	0.635 (0.04)	0.643 (0.04)	−0.010 (0.03)	3/36
	APA	CO	2011/2012	38	2.974 (0.34)	4.91 (0.27)	0.605 (0.07)	0.617 (0.05)	0.626 (0.05)	0.046 (0.05)	0/36
	APP	TT	2011/2012	39	3.008 (0.35)	5.19 (0.3)	0.631 (0.04)	0.622 (0.05)	0.631 (0.05)	−0.028 (0.03)	1/36
	RP	PT	2011/2012	14	3.270 (0.45)	4.69 (0.09)	0.643 (0.07)	0.648 (0.05)	0.672 (0.05)	0.033 (0.06)	0/36
	SA	TE	2011/2012	61	2.871 (0.20)	5.28 (0.3)	0.627 (0.04)	0.631 (0.04)	0.639 (0.04)	0.008 (0.02)	3/36
South	APA	V1	2010/2011	46	2.971 (0.34)	4.82 (0.19)	0.595 (0.04)	0.623 (0.05)	0.630 (0.05)	0.037* (0.04)	2/36
	APP	V2	2010/2011	23	2.991 (0.32)	4.56 (0.17)	0.663 (0.05)	0.627 (0.05)	0.649 (0.05)	−0.069 (0.06)	0/36
	APC	V1	2010/2011	15	2.820 (0.29)	4.68 (0.15)	0.633 (0.04)	0.615 (0.04)	0.629 (0.04)	−0.031 (0.05)	1/36
	APA	U	2010/2011	98	3.186 (0.35)	5 (0.3)	0.689 (0.04)	0.650 (0.04)	0.653 (0.04)	−0.065 (0.02)	4/36
	APA	PN	2011/2012	64	3.040 (0.33)	5.01 (0.32)	0.631 (0.06)	0.633 (0.05)	0.638 (0.05)	0.014 (0.04)	10/36
	APP	VP	2011/2012	30	2.819 (0.29)	4.94 (0.29)	0.582 (0.05)	0.609 (0.05)	0.620 (0.05)	0.041 (0.04)	3/36
	RP	PI	2011/2012	12	2.852 (0.34)	4.55 (0)	0.620 (0.05)	0.600 (0.05)	0.626 (0.06)	−0.052 (0.04)	0/36
	SA	PI	2011/2012	55	3.066 (0.30)	5.24 (0.33)	0.620 (0.04)	0.639 (0.04)	0.646 (0.05)	0.029 (0.02)	1/36
	Total			676	3.007 (0.08)	4.88 (0.23)	0.632 (0.01)	0.631 (0.01)	0.641 (0.01)	−0.003 (0.01)	5/36

*N*: number of multilocus genotypes; *N*_e_: number of effective alleles; A: standardized allelic richness; *H*_o_: observed heterozygosity; *H*_e_: expected heterozygosity; u*H*_e_: unbiased expected heterozygosity; F_IS_: inbreeding coefficient and LD: linkage disequilibrium. (SD): standard deviation; (SE): standard error

* indicates the *P*-value for *F*_IS_ that are statistically different from zero. APA: *Acyrthosiphon pisum* alfalfa race; APP: *A. pisum* pea race; APC: *A. pisum* red clover race; SA: *Sitobion avenae*; RP: *Rhopalosiphum padi*.

#### Reconstructing sibship relationships among parasitoid genotypes within and between populations

To study weak genetic differentiations and compare male and female related gene flows of *A. ervi* between populations, a sibship reconstruction (full-sibship and half-sibship) without prior knowledge of the parental genotypes was carried out using the full-likelihood method implemented in COLONY for haplodiploid species (Jones and Wang 2010). This method has been shown to be especially useful when populations exhibit a weak genetic structure due to high migration rates, and where *F*_ST_ and other methods have a limited resolution to detect genetic differentiation (Jones and Wang 2012). In this sense, when populations are unstructured, the relatedness (e.g., sibship dyads) will be distributed randomly among subpopulations; but in structured populations, sibship is more likely to be found within a particular subpopulation than between subpopulations, then by comparing the average relatedness within and between subpopulations is possible to infer the population structure (Piyapong et al. 2011; Jones and Wang 2012). Two analyses were carried out separately, one including genotypes from four parasitoid populations sampled on a particular aphid host/site in the Maule Region (populations: APA (CO) and APP (TT) races of *A. pisum*,*S. avenae* (TE) and *R. padi* (PT); Fig.2) (distance range between populations was 2–20 km), and other using genotypes sampled from three different populations in the Los Ríos Region (populations: APA (PN) and APP (VP) races of *A. pisum* and *S. avenae* -*R. padi* (PI); Fig.2) (distance range between hosts was 40–69 km). In this latter analysis, parasitoid genotypes from the aphid hosts *S. avenae* and *R. padi* were treated as a single population for the study of the distribution of sibships, because parasitoids were sampled on both aphid species in a same field and time. To limit the chance of taking individuals from more than two different generations (the method assumes a single generation), only individuals sampled in the same month were included. In addition, only genotypes from populations sampled during the 2011/2012 season were used in the analyses, as genotypes from different populations were not sampled within the same month in the season 2010/2011. In the analyses, we assumed a model of monogamous females (i.e., single mating for females) and polygamous males (i.e., multiple mating for males) without inbreeding, since our results did not show any evidence of inbreeding (see Results). Thus, full-sib and paternal half-sib relationships were estimated. This type of reproductive strategy has been reported for *A. ervi* (He and Wang 2008) and previously used for kinship analysis in aphid parasitoids (see Tentelier et al. 2008). The frequency and distribution of full-sibs (shared by both parents) and paternal half-sib pairs (sharing only the same father) was studied within (sibship dyads present on a same parasitoid population) and between (sibship dyads present on different parasitoid populations) parasitoid populations. To better estimate kinship coefficients and to enhance the probability of finding the best configuration with the maximum likelihood within each dataset, an inferred genotyping error rate for all loci was assumed (using COLONY), including multiple runs (*n* = 10) with a long length of run (Jones and Wang 2010). Only sibship dyads with a probability ≥ 0.9 from the analyses were considered. Differences on the distribution of full-sib and paternal half-sib frequencies within and between different parasitoid populations (H0: equally distribution of full-sib and half-sib dyads within and between parasitoid populations) were tested using Fisher's exact tests for contingency tables in R (R Development Core Team 2011). The correlation between the observed proportion of paternal half-sib dyads within and between the parasitoid populations (standardized by the total of possible dyads; full-sib, half-sib, and nonrelated dyads) in each analysis, and the geographic distance between parasitoid populations was studied using Pearson's correlation in R (R Development Core Team 2011). Finally, an analysis including all genotypes analyzed from both regions was performed to identify the possibility of false positives (i.e., sibship dyads between regions) into sibship inferences.

## Results

### Genetic diversity within parasitoid populations

A total of 676 parasitoid females sampled from 15 populations of different aphid hosts, locations, and seasons were analyzed with nine microsatellite loci. Each individual represented a unique multilocus genotype. The genetic diversity within each parasitoid population was similar, with an observed heterozygosity ranging from 0.582 to 0.659 and an expected heterozygosity ranging from 0.566 to 0.673 (Table2). Likewise, the standardized allelic richness also was similar for all populations (Table2). Overall, few loci were found in linkage disequilibrium; 10 of 36 pairs of loci were found in LD for parasitoid populations coming from *A. pisum* alfalfa race in the Los Ríos Region (sampled in 2011/2012 season; Table2), and only one parasitoid population (from *A. pisum* alfalfa race in the La Araucanía Region, sampled in 2010/2011 season) showed significant deviations from HWE due to homozygote excess. All the other parasitoid populations were found to be in HWE (Table2).

### Genetic differentiation among parasitoid populations

**Figure 3:**
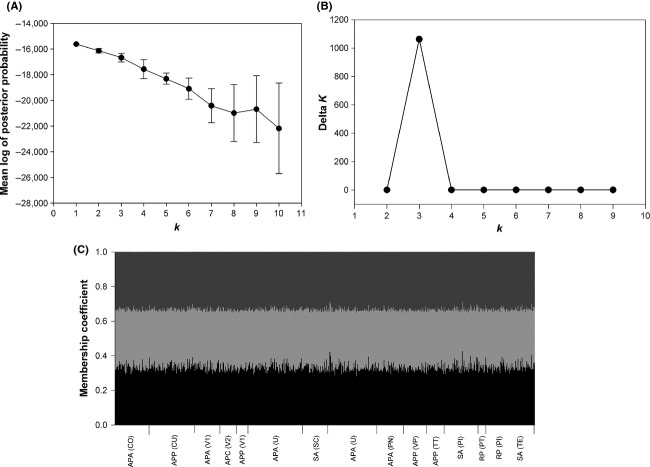
Bayesian assignment analysis for each genotype found in this study. The multilocus genotypes sampled from different aphid hosts (APA:*A. pisum* alfalfa; APP:*A. pisum* pea; APC:*A. pisum* clover; SA:*S. avenae*, and RP:*R. padi*) and localities (localities codes detailed in Figure1) in the two regions studied and during two consecutive seasons 2010/2011 (1–7 populations) and 2011/2012 (8–15 populations) were analyzed. (A) Mean log of posterior probability for each number of clusters (*k*) (mean ± SD); (B) Change rate of the posterior probability (Delta *k*) for different numbers of clusters (*k*); (C) Structure plot for *k* = 3 shows the proportion of each multilocus genotype assigned to each of the estimated cluster (membership coefficients).

Our results revealed a weak genetic differentiation and a lack of genetic structure among parasitoid populations from different aphid host species/races and geographic regions. The nine microsatellite loci and sample sizes of populations under study were found to be diverse enough to detect genetic differentiation between populations with a *F*_ST_ as low as 0.0025 with a high overall resolution power (0.95) and with a low risk of false significances (*α *< 0.05), according to the POWSIM analyses (Ryman and Palm 2006). This latter analysis ruled out the possibility of making a type-II error. The AMOVA showed a nonsignificant genetic variation among aphid hosts, regions, or seasons (Table3). Consequently, parasitoid populations from different aphid hosts and different regions showed a low genetic differentiation for the 2010/2011 season data (low *F*_ST_ and 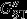 values between pairs of populations). Only the parasitoid populations from *S. avenae* in Maule Region and *A. pisum* red clover race in La Araucanía Region showed a small but significant genetic differentiation (*F*_ST_ = 0.014, *P* < 0.05; 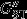 = 0.028, *P* < 0.05) (Table S2a). Similarly, a low genetic differentiation between parasitoid populations was observed for the data sampled in 2011/2012 season, with the exception of a low but significant genetic differentiation between parasitoid populations from *S. avenae* compared to *A. pisum* alfalfa race in the Maule Region (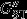 = 0.020; *P* < 0.05; *F*_ST_ = 0.010, *P* < 0.05) and from *A. pisum*-alfalfa race in the Los Ríos Region (*F*_ST_ = 0.027, *P* < 0.05; 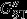 = 0.010, *P* < 0.05) (Table S2b). The Bayesian clustering analysis also failed to detect any genetic structure when all multilocus genotypes of *A. ervi* from different aphid hosts, regions, or seasons were included. The genotypes were not grouped into a *k* ≥ 1 cluster, being all multilocus genotypes assigned to one single genetic cluster (i.e., ∽1/number of clusters) in a similar proportion (membership coefficient), thus supporting the lack of genetic structure described above using AMOVA (Fig.3C). The highest average posterior probability was obtained for *k* = 1, and the highest rate of change (delta *k*) was for *k* = 3 (Fig.3A and B); however, the graphical input clearly shows that all the individuals are descendent from a single population (all individuals descend from the same three clusters, without any trend).

**Table 3:** Hierarchical analysis of the molecular variance (AMOVA) in parasitoid populations according to the aphid host, sampled regions and seasons

Source	df	Variance	*F* value	*P* value
Among aphid hosts	4	0.002	0.001	0.443
Among regions within a host	10	0.002	0.001	0.242
Within populations	1337	2.898	0.002	0.213
Total	1351	2.903		
Among seasons	1	0.002	0.001	0.189
Among populations within seasons	13	0.002	0.001	0.284
Within populations	1337	2.898	0.001	0.167
Total	1351	2.902		
Among regions	1	0.002	0.001	0.889
Among populations within region	14	0.003	0.001	0.134
Within populations	1336	2.898	0.002	0.268
Total	1351	2.904		

df: degrees of freedom.

### Relationships among parasitoid individuals within and between populations

**Figure 4:**
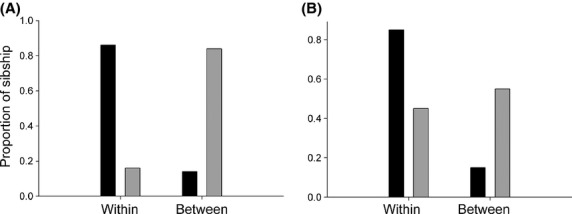
Distribution of the Sibship proportion within and between parasitoid populations for (A) Maule Region and (B) Los Ríos Region. The total of full-sib dyads (black) and paternal half-sib dyads (light gray) has been graphed.

We studied the frequency and distribution of full-sib and half-sib pairs using the multilocus genotypes of *A. ervi* females from different populations sampled on *A. pisum* alfalfa and pea races, *S. avenae* and *R. padi*. The sibship analyses identified a total of 20 full-sib dyads, 96 half-sib dyads, and 9754 nonsibship dyads among the 141 multilocus genotypes of *A. ervi* analyzed from Los Ríos Region (southern). In the Maule Region (central), seven full-sib dyads, 49 half-sib dyads, and 4600 nonsibship dyads were observed among the 97 multilocus genotypes analyzed. The results show that the full-sib frequency was higher within (17 full-sib dyads) than between parasitoid populations (three full-sib dyads) in Los Ríos Region, as well as in the Maule Region, where the full-sib frequency was higher within (six full-sib dyads) than between populations (only one full-sib dyad) (Fig.4). Contrastingly, for both geographic regions, the frequency of paternal half-sibs was higher between (53 half-sib in Los Ríos Region and 41 half-sib in the Maule Region) than within populations (43 half-sib in Los Ríos Region and eight half-sib in the Maule Region) (Fig.4). This differential distribution of paternal half-sibs and full-sibs across different populations was consistent and significant in both regions: Los Ríos (*F* exact test, *odds ratio* = 0.14; *P *=* *0.001) and Maule (*F* exact test, *odds ratio* = 0.035; *P *=* *0.0005) (Fig.4).

It is also interesting to note that the frequency of paternal half-sibs observed between populations associated with host races (APA -APP) of *A. pisum* (intraspecific level) was lower than the paternal half-sib frequency observed between the populations associated with different aphid species (e.g., APA-SA or APP-SA) (Table S3). This was true in spite of that the distance (km) between parasitoid populations sampled from different host races (APA - APP) was less than the distance between parasitoid populations sampled from different aphid species (APA-SA or APP-SA) in both studied regions (Maule and Los Rios regions) (Table S3).

In fact, there was a nonsignificant correlation between the proportion of paternal half-sib dyads of parasitoids (numbers of half-sib dyads/total of possible dyads by analysis) and the geographic distance between parasitoid populations (*r* = 0.4; df = 14; *P* value = 0.08), including comparisons between the two regions (Table S3). Finally, an additional analysis including all genotypes from the two sibships analyses (different regions) was conducted. This method inferred sibship dyads within regions in a proportion of 0.61 among the total of inferred sibships (242 sibship dyads). However, sibship dyads were also observed between regions in a proportion of 0.38, suggesting the possibility of some false-positive assignments in the analyses, as some sibship dyads were observed between genotypes sampled from different geographic regions (∽500 km apart), which is highly unlikely even for a good disperser insect.

## Discussion

### Lack of genetic differentiation among populations

The results in the present work support a lack of genetic differentiation among populations, since the genetic diversity of *A. ervi* parasitoids in Chile appears homogeneously distributed in space and time among different aphid hosts, providing evidence of a large panmictic population of *A. ervi* in Chile. The lack of genetic structuring was irrespective of the host-aphid species/races on which parasitoids were sampled, even between different regions located apart more than 400 km, and between two consecutive seasons. The absence of differentiation presumably resulted from the highly homogenizing effect of parasitoid dispersal driving a strong gene flow among populations. Furthermore, dispersal abilities of parasitoids could be further increased when a proportion of the parasitized aphid hosts is able to colonize new patches, passively carrying parasitoid larvae, enabling a greater dispersion of these parasitic wasps (Feng et al. 2007). This latter phenomenon has been reported for *A. ervi* and *A. rhopalosiphi* in winged aphid migrants of *S. avenae* (Walton et al. 2011). Our results are concordant with Bilodeau et al. (2013), which found a lack of host-associated parasitoid populations in introduced populations of *A. ervi* associated with two specialized host races of the pea aphid *A. pisum* (alfalfa and red clover host races) in a smaller scale study (between 160–240 km) in North America, using a different set of microsatellites. In addition, other several studies on aphid parasitoids have shown a lack of host-associated genetic differentiation (Lozier et al. 2009; Dickey and Medina 2011; Lavandero et al. 2011).

It should be noted that both studies in *A. ervi* included introduced populations for classic biological control (in Chile and North America), which could be influenced by insufficient time for host-associated selective agents resulting in adaptive divergence in these parasitoid populations (Bilodeau et al. 2013).

### Differential dispersal of parasitoid females and males

The results of the sibship analyzes carried out here showed significant differences in the distribution of full-sib and paternal half-sib dyads within and between different parasitoid populations in the field. A higher frequency of full-sibs was observed within populations in relation to that observed between populations. This observation could be the result of host fidelity in *A. ervi* females, as parasitoid populations were sampled on different aphid hosts in the field. Therefore, female parasitoids exhibiting a preference to oviposit on the same aphid host species from where they emerged should show a lower dispersal to other host-associated populations (Feder et al. 1994). Other nonmutually exclusive explanations come from the fact that host-patch foraging could strongly affect the reproductive success of parasitoid females due to the higher energy costs and the higher mortality risks associated with dispersal (Weisser et al. 1994). Interestingly, the results show that the frequency of paternal half-sibs was greater between populations than within themselves. In unstructured populations, sibship dyads should be expected to be randomly distributed among subpopulations, while if subpopulations are structured, sibships will be more likely found within than between populations (Jones and Wang 2012). Thus, when migration increases, sibships within subpopulations will tend to decrease relative to that between subpopulations (Jones and Wang 2012). Thereby, a higher paternal half-sib proportion between populations might suggest that *A. ervi* males are more likely to disperse, acting as mediators of gene flow between parasitoid populations.

Sibship analysis implemented in COLONY represents a powerful approach to study weak-structured populations in relation to other similar methods, even at higher genotyping error rates (Wang 2004; Harrison et al. 2013a,b). It has been proved to be useful to investigate sex-biased dispersal in haplodiploid Hymenoptera, because the haploid nature of males causes a high relatedness among sibs (Lepais et al. 2010). However, the possibility of false-positive assignments needs to be considered in this type of analysis (Christie 2013; Harrison et al. 2013b), being the number and diversity of loci some of the main factors affecting the accuracy of the method (Harrison et al. 2013b). Even when the number of loci seems appropriate to achieve a suitable level of certainly in sibship inferences (Wang 2004; Wang and Santure 2009). An additional analysis including parasitoid genotypes from both regions revealed the possibility of false-positive assignments in a proportion of 0.38 in our study. This could be due to the uneven observed allele frequency in the dataset, where some unique alleles were observed in high frequencies (>0.4) in most of the loci studied (eight of nine loci) (Table S4). Hence, an unequal allele frequency may decrease the sibship exclusion probability (i.e., the average capability of the markers to exclude unrelated individuals from the sibship relationships), which reach its maximum when all the alleles have the same frequency (Wang 2007). In this regard, sibships reconstructed from analyses should be interpreted with caution; being utilized as a method to study weak genetic structure, as done herein for the studied *A. ervi* populations, instead of estimating the actual pedigree relationships.

Regarding the higher paternal half-sibs between than within parasitoid populations, several interacting factors could contribute to a greater dispersion of *A. ervi* males between populations. (1) A male-biased sex ratio (Clobert et al. 2001; Ronce 2007; Nelson and Greeff 2011), though sex ratio is commonly female-biased in most aphid parasitoid species (Mackauer 1976; Sequeira and Mackauer 1993); (2) A local mate competition between males (Nelson and Greeff 2011; Greeff et al. 2003; Henry 2008), which has already been described for *Aphidius* parasitoids (Godfray 1994); (3) A reproductive strategy resulting in monogamous mating for *A. ervi* females and polygamous males (Godfray 1994; He and Wang 2008); (4) The existence of philopatry in female parasitoids (Johnstone et al. 2012) or host fidelity in females of *A. ervi* (Zepeda-Paulo et al. 2013); and (5) the occurrence of protandry, in which male parasitoids emerge from their mummies before female parasitoids, a phenomenon described for several parasitoid species including *A. ervi* (Godfray 1994; He et al. 2004). Therefore, males should be more likely to leave the natal host patch looking for suitable mates elsewhere when females and/or virgin females are absent, which is common when females exhibit a monogamous behavior (Martel et al. 2008). Indeed, Nyabuga et al. (2012) reported differences between sexes of *A. ervi* in terms of dispersal behavior after emergence; they found that the residence time on the natal patch is shorter in males than in females*,* encouraging male dispersion as a putative strategy to reduce competition between males. Furthermore, male competition for mating has been reported to be strongly influenced by male body size in *A. ervi*, larger males having a competitive advantage over smaller ones (Henry 2008). In *Aphidius* parasitoids, body size is a highly plastic trait partially determined by the size of their natal host (Godfray 1994). Hence, parasitoid populations reproducing on larger natal hosts (e.g., *A. pisum* host races) could limit the gene flow from parasitoids immigrating from smaller hosts (e.g., *S. avenae* or *R. padi*) due to competitive advantages of larger males. This suggests that the natal host of males should influence their ability to mate with females coming from neighboring populations on different aphid hosts (Henry 2008). This could account for the lower frequency of paternal half-sib dyads observed between hosts races of *A. pisum* (APP and APA) (the larger host) compared with the higher frequency of paternal half-sib dyads observed between *A. pisum* and *S. avenae* (the smaller host) (APP-SA or APA-SA), even when the geographic distances between samples from different aphid species (APP-SA or APA-SA) are greater than between samples from distinct host races (APP-APA) (for both geographic regions analyzed).

Patterns of sex-biased dispersal have been frequently observed among certain birds, mammals, and hymenopteran social insects (e.g., bees and ants) (Hedrick 2007; Johnstone et al. 2012). However, male-mediated dispersion has not been reported and/or studied so far in hymenopteran parasitoid insects, although it has been proposed as a plausible mechanism relevant to the processes of host adaptation in parasitoids (Henry 2008). As a consequence, a high gene flow between environments (different hosts) would be detrimental to local adaptation (Kawecki and Ebert 2004) and host-associated genetic differentiation, due to its homogenizing effect between parasitoid populations exploiting different hosts (Abrahamson and Blair 2008).

## Conclusions

The present study reports evidence of a high gene flow among parasitoid populations on different aphid hosts and geographic regions, resulting in a lack of genetic differentiation in the natural populations of *A. ervi*. Interestingly, the pattern of dispersal and gene flow between parasitoid populations seems to be asymmetrical for males and females, suggesting that females should have a rather restricted dispersal between populations, while males would be the main drivers of gene flow between parasitoid populations. Male-driven gene flow could represent a significant factor impeding the formation of host-associated genetic differentiation and local host adaptation in *Aphidius* parasitoids. Finally, more studies focused on sex-biased dispersal in parasitoid species using controlled experiments to examine the potential of parasitoid males as a source of dispersal and gene flow between different hosts are needed to confirm the dispersal pattern observed in this study and its effect on host-associated genetic differentiation and host adaptation in parasitoid species.

## Data Accessibility

The following raw data supporting the results of this article have been deposited in the Dryad repository. doi:10.5061/dryad.13mn3.

The multilocus genotype for each sampled individual.
The input files used for Sibship inferences in COLONY.
GenBank accession numbers of microsatellite loci.

## Supporting Information

**Table S1**. Novel polymorphic microsatellite loci developed for the aphid parasitoid *Aphidius ervi*. **Table S2**. (a) Genetic differentiation between pairs of parasitoid populations according to the host-aphid and region sampled during 2010/2011 season. (b) Genetic differentiation between pairs of parasitoid populations according to the host-aphid and region sampled during 2011/2012 season. **Table S3**. Distribution of paternal half-sibs within and between different parasitoid populations sampled on *A. pisum* alfalfa (APA), *A. pisum* pea (APP), *S. avenae* (SA) and *R. padi* (RP) host species. **Table S4**. Allele frequencies at nine microsatellite loci of *A. ervi*.
