# Supplementary material for: Does sex-biased dispersal account for the lack of geographic and host-associated differentiation in introduced populations of an aphid parasitoid?
Source: Ecol Evol. 2015 May 6;5(11):2149–61. doi: 10.1002/ece3.1504 (PMC4461417; doi:10.1002/ece3.1504)
Supplement: Supplementary file 1 [file ece30005-2149-sd1.docx]

**Additional file 1**

**Table S1** Novel polymorphic microsatellite loci developed for the aphid parasitoid *Aphidius ervi*. Primer sequences (F: forward; R: reverse). Allele sizes. Number of different alleles (Na). Number of effective alleles (Ne). Observed heterozygosity (Ho). Expected heterozygosity (He). Unbiased heterozygosity (uHe). Inbreeding coefficient (Fis) and its p-value are all described for each microsatellite locus. * Indicates the p-value for Fis that are statistically different from zero.

| **Locus** | **Sequence (5'-3')** | **Allele size** | **Na** | **Ne** | **Ho** | **He** | **uHe** | **Fis** | ***P* value** |
| --- | --- | --- | --- | --- | --- | --- | --- | --- | --- |
| Ae01 | F: CAGTGGACCACAATCACCTG | 190-220 | 4 | 3.57 | 0.717 | 0.720 | 0.721 | 0.005 | 0.1048 |
|  | R: TCATTGTCGGAATTTTGATGA |  |  |  |  |  |  |  |  |
| Ae03 | F: TGAGCAACAATCCGAAACTG | 140-180 | 15 | 2.30 | 0.551 | 0.566 | 0.566 | 0.027 | 0.0363* |
|  | R: CCAATAATATAAGAGAAGCAAACG |  |  |  |  |  |  |  |  |
| Ae06 | F: ACCACCACTATCATCAGC | 190-230 | 6 | 3.17 | 0.688 | 0.685 | 0.685 | -0.004 | 0.9458 |
|  | R: AAAGGTGTGATTCCAAAGTG |  |  |  |  |  |  |  |  |
| Ae08 | F: TCCTTTGGTAAATGCTTGGA | 110-170 | 7 | 3.38 | 0.683 | 0.704 | 0.704 | 0.029 | 0.0726 |
|  | R: ATAACAGTGGTGTGCCCCTC |  |  |  |  |  |  |  |  |
| Ae16 | F: AACGCCGAAATTTTCATTTG | 280-310 | 4 | 1.70 | 0.419 | 0.412 | 0.412 | -0.018 | 0.9106 |
|  | R: CCAGTGGTTTGTAAATTACTTGATG |  |  |  |  |  |  |  |  |
| Ae20 | F: TTGGATCTGCTTGAGGGTCT | 110-145 | 10 | 3.17 | 0.607 | 0.685 | 0.685 | 0.113 | 0.0000* |
|  | R: TCTCCTCTTCTTTCTCTGCTTCA |  |  |  |  |  |  |  |  |
| Ae22 | F: TGTACAAGTGACAGTAGTAACAACAA | 90-130 | 7 | 3.95 | 0.532 | 0.747 | 0.748 | 0.288 | <0.0000* |
|  | R: ATAGCAGGCCAGACAATGCT |  |  |  |  |  |  |  |  |
| Ae27 | F: AGCACCAAATCAAGCAACAGT | 195-240 | 6 | 1.98 | 0.493 | 0.494 | 0.494 | 0.001 | 0.5399 |
|  | R: CTGGTATTGTTGAATTTGAATGA |  |  |  |  |  |  |  |  |
| Ae29 | F: TTCGAACATGTCGTGTCCAT | 240-290 | 11 | 4.12 | 0.728 | 0.757 | 0.758 | 0.039 | 0.1785 |
|  | R: CGACTCACACTCACCCTCCT |  |  |  |  |  |  |  |  |
| Ae32 | F:GAAAATATAGAGAGGGAGAAAAAGAGAA | 160-180 | 6 | 3.30 | 0.691 | 0.697 | 0.697 | 0.008 | 0.5077 |
|  | R: CCATTCAATGACGATACCCC |  |  |  |  |  |  |  |  |
| Ae33 | F: TTGCTGTTGTACAATTAGCTGG | 90-125 | 13 | 4.21 | 0.780 | 0.763 | 0.763 | -0.022 | 0.3086 |
|  | R: TCACAAAAATCCATGACTAATAA |  |  |  |  |  |  |  |  |
| Ae38 | F: CATCTTCATTATCAACAACAGCA | 100-120 | 5 | 3.61 | 0.356 | 0.723 | 0.724 | 0.507 | <0.0000* |
|  | R: TCAAGAAAATTCAAAAGTATCAAA |  |  |  |  |  |  |  |  |

**Additional file 2**

**Table S2a** Genetic differentiation between pairs of parasitoid populations according to the host-aphid and region sampled during 2010/2011 season. Fst values are in the lower diagonal and G''st values in the above diagonal. Asterisk indicates significance at *P* < 0.05. APA: *Acyrthosiphon pisum* race alfalfa; APP: *A. pisum* race pea; APC: *A. pisum* race red clover; SA: *Sitobion avenae*; RP: *Rhopalosiphum padi.*

| **Parasitoid population** | **APA-Central** | **APP-Central** | **SA-Central** | **APA1-South** | **APC-South** | **APP-South** | **APA-South** |
| --- | --- | --- | --- | --- | --- | --- | --- |
| **APA-Central** | - | -0.002 | -0.001 | -0.006 | 0.000 | 0.008 | 0.009 |
| **APP-Central** | 0.004 | - | -0.010 | 0.004 | 0.010 | -0.010 | 0.004 |
| **SA-Central** | 0.006 | 0.003 | - | 0.000 | 0.028* | -0.009 | 0.007 |
| **APA1-South** | 0.004 | 0.005 | 0.006 | - | -0.002 | -0.015 | 0.001 |
| **APC-South** | 0.008 | 0.009 | 0.014* | 0.008 | - | 0.005 | 0.014 |
| **APP-South** | 0.012 | 0.008 | 0.010 | 0.009 | 0.015 | - | -0.005 |
| **APA-South** | 0.005 | 0.003 | 0.005 | 0.004 | 0.009 | 0.008 | - |

**Table S2b** Genetic differentiation between pairs of parasitoid populations according to the host-aphid and region sampled during 2011/2012 season. Fst values are in the lower diagonal and G''st values in the above diagonal. Asterisk indicates significant at P < 0.05. APA: *Acyrthosiphon pisum* race alfalfa; APP: A. pisum race pea; APC: *A. pisum* race red clover; SA: *Sitobion avenae*; RP: *Rhopalosiphum padi*.

| **Parasitoid population** | **APA Central** | **APP Central** | **SA Central** | **RP Central** | **APA South** | **APP South** | **SA South** | **RP South** |
| --- | --- | --- | --- | --- | --- | --- | --- | --- |
|  |  |  |  |  |  |  |  |  |
| **APA Central** | - | -0.021 | 0.020* | 0.022 | 0.004 | 0.007 | 0.003 | 0.012 |
|  |  |  |  |  |  |  |  |  |
| **APP Central** | 0.003 | - | 0.016 | 0.033 | 0.003 | 0.008 | 0.004 | 0.013 |
|  |  |  |  |  |  |  |  |  |
| **SA Central** | 0.010 | 0.009 | - | 0.030 | 0.010* | 0.010 | 0.006 | 0.015 |
|  |  |  |  |  |  |  |  |  |
| **RP Central** | 0.017 | 0.019 | 0.017 | 0.00 | 0.015 | 0.015 | 0.013 | 0.028 |
|  |  |  |  |  |  |  |  |  |
| **APA South** | -0.010 | -0.012 | 0.027* | 0.022 | - | -0.010 | -0.011 | -0.012 |
|  |  |  |  |  |  |  |  |  |
| **APP South** | -0.005 | -0.003 | 0.014 | 0.001 | 0.005 | - | -0.016 | 0.004 |
|  |  |  |  |  |  |  |  |  |
| **SA South** | -0.016 | -0.013 | 0.003 | 0.004 | 0.003 | 0.005 | - | -0.016 |
|  |  |  |  |  |  |  |  |  |
| **RP South** | -0.010 | -0.004 | 0.009 | 0.044 | 0.011 | 0.016 | 0.010 | - |

**Additional file 3**

**Table S3** Distribution of paternal half-sibs within and between different parasitoid populations sampled on *A. pisum*-alfalfa (APA), *A. pisum*-pea (APP), *S. avenae* (SA) and *R. padi* (RP) host species. Number of genotypes in the different populations (n) and distance (km) between parasitoid populations. Frequency and proportion of paternal half-sib dyads (standardized by the total of possible dyads; full-sibs, half-sibs and unrelated) within and between the parasitoid populations for each geographic region are detailed.

| Maule Region | | | | | Los Rios Region | | | | |
| --- | --- | --- | --- | --- | --- | --- | --- | --- | --- |
|  | **n** | **Half-sibs** | **Proportion** | **Km** |  | **n** | **Half-sibs** | **Proportion** | **Km** |
| Within |  |  |  |  | **Within** |  |  |  |  |
| APA | 12 | 0 | 0 | 0 | APA | 42 | 16 | 0.0016 | 0 |
| APP | 35 | 4 | 0.0009 | 0 | APP | 26 | 1 | 0.0001 | 0 |
| SA | 41 | 3 | 0.0007 | 0 | SA_RP | 73 | 26 | 0.0027 | 0 |
| RP | 9 | 1 | 0.0002 | 0 |  |  |  |  |  |
| Total |  | 8 (16%) |  |  | Total |  | 43 (45%) |  |  |
| Between |  |  |  |  | **Between** |  |  |  |  |
| APA-APP |  | 4 | 0.0009 | 2 | APA-APP |  | 7 | 0.0007 | 21 |
| APA-SA |  | 7 | 0.0015 | 19 | APA-SA_RP |  | 20 | 0.0020 | 69 |
| APP-SA |  | 17 | 0.0037 | 17 | APP-SA_RP |  | 26 | 0.0027 | 57 |
| RP-SA |  | 7 | 0.0015 | 28 |  |  |  |  |  |
| RP-APP |  | 4 | 0.0009 | 17 |  |  |  |  |  |
| RP-APA |  | 2 | 0.0004 | 17 |  |  |  |  |  |
| Total |  | 41 (84%) |  |  | Total |  | 53 (55%) |  |  |

**Additional file 4**

**Table S4** Allele frequencies at nine microsatellite loci of *A. ervi*.

| **Locus** | **Ae01** | | **Ae03** | | **Ae06** | | **Ae08** | | **Ae16** | | **Ae27** | | **Ae29** | | **Ae32** | | **Ae33** | |
| --- | --- | --- | --- | --- | --- | --- | --- | --- | --- | --- | --- | --- | --- | --- | --- | --- | --- | --- |
| N° of alleles | Alleles | F | Alleles | F | Alleles | F | Alleles | F | Alleles | F | Alleles | F | Alleles | F | Alleles | F | Alleles | F |
| 1 | 201 | 0.396 | 151 | 0.640 | 209 | 0.089 | 135 | 0.031 | 298 | 0.722 | 204 | 0.132 | 276 | 0.156 | 161 | 0.041 | 104 | 0.026 |
| 2 | 204 | 0.193 | 159 | 0.012 | 213 | 0.112 | 138 | 0.018 | 301 | 0.257 | 209 | 0.688 | 279 | 0.022 | 163 | 0.055 | 107 | 0.080 |
| 3 | 207 | 0.180 | 161 | 0.115 | 215 | 0.009 | 145 | 0.001 | 310 | 0.012 | 213 | 0.091 | 283 | 0.060 | 167 | 0.432 | 110 | 0.226 |
| 4 | 210 | 0.231 | 163 | 0.066 | 218 | 0.404 | 148 | 0.128 | 316 | 0.009 | 215 | 0.086 | 287 | 0.435 | 169 | 0.287 | 113 | 0.034 |
| 5 |  |  | 166 | 0.001 | 221 | 0.362 | 151 | 0.445 |  |  | 218 | 0.002 | 289 | 0.097 | 171 | 0.173 | 116 | 0.406 |
| 6 |  |  | 170 | 0.037 | 225 | 0.024 | 155 | 0.258 |  |  | 221 | 0.002 | 290 | 0.038 | 173 | 0.011 | 119 | 0.082 |
| 7 |  |  | 173 | 0.002 |  |  | 161 | 0.119 |  |  |  |  | 294 | 0.002 |  |  | 126 | 0.002 |
| 8 |  |  | 175 | 0.002 |  |  |  |  |  |  |  |  | 296 | 0.023 |  |  | 129 | 0.052 |
| 9 |  |  | 177 | 0.052 |  |  |  |  |  |  |  |  | 298 | 0.090 |  |  | 132 | 0.059 |
| 10 |  |  | 180 | 0.002 |  |  |  |  |  |  |  |  | 300 | 0.077 |  |  | 135 | 0.002 |
| 11 |  |  | 182 | 0.002 |  |  |  |  |  |  |  |  | 302 | 0.001 |  |  | 138 | 0.001 |
| 12 |  |  | 186 | 0.001 |  |  |  |  |  |  |  |  |  |  |  |  | 143 | 0.011 |
| 13 |  |  | 190 | 0.062 |  |  |  |  |  |  |  |  |  |  |  |  | 145 | 0.020 |
| 14 |  |  | 192 | 0.003 |  |  |  |  |  |  |  |  |  |  |  |  |  |  |
| 15 |  |  | 194 | 0.004 |  |  |  |  |  |  |  |  |  |  |  |  |  |  |
|  |  |  |  |  |  |  |  |  |  |  |  |  |  |  |  |  |  |  |

F: allele frequency
